# Comparison of visual and semi‐automated kilovoltage cone beam CT image QA analysis

**DOI:** 10.1002/acm2.14190

**Published:** 2023-11-08

**Authors:** Nicholas Becerra‐Espinosa, Lindsey Claps, Parham Alaei

**Affiliations:** ^1^ Department of Radiation Oncology University of Minnesota Minneapolis Minnesota USA; ^2^ Present address: Northwestern Medicine Proton Center, 4455 Weaver Pkwy Warrenville, IL 60555 USA; ^3^ Present address: Department of Medical Physics Memorial Sloan Kettering Cancer Center, 1275 York Avenue New York, NY 10065 USA

**Keywords:** image QA, kilovoltage cone‐beam computed tomography

## Abstract

Established kilovoltage cone‐beam computed tomography (kV‐CBCT) image quality assurance (QA) guidelines often rely on recommendations provided by the American Association of Physicists in Medicine (AAPM) task group (TG) reports with metrics that use visual analysis. This can result in measurement variations by different users, especially in visually subjective analyzes such as low contrast resolution. Consequently, there is a growing interest in more automated means of image QA analysis that can offer increased consistency, accuracy, and convenience. This work compares visual QA to semi‐automated software QA analysis to establish the performance and viability of a semi‐automated method.

In this study, a commercial product (RIT Radia. Radiological Imaging Technology, Colorado Springs, CO) was used to evaluate 68 months of kV‐CBCT images of a Catphan**®** 504 phantom obtained from a Varian TrueBeam**®** linear accelerator. Six key metrics were examined: high contrast resolution, low contrast resolution, Hounsfield unit constancy, uniformity and noise, and spatial linearity. The results of this method were then compared to those recorded visually using Bland‐Altman, and/or paired sample t‐test.

Comparison of all modules showed a non‐random, statistically significant difference between visual and semi‐automated methods except for LDPE and Teflon in the Hounsfield unit constancy analysis, which falls outside the paired sample *t*‐test's 5% significance level. A small high contrast resolution bias indicates the two analysis methods are largely equivalent, while a large low contrast resolution bias indicates greater semi‐automated target detection. Wide limits of agreement in the uniformity module suggests variability due to multiple visual observers. Spatial linearity results measured differences of less than 0.17%.

Semi‐automated QA analysis offered greater stability over visual analysis. Additionally, semi‐automated QA results satisfied or exceeded visual QA passing criteria and allowed for fast and consistent image quality analysis.

## 1 INTRODUCTION

Routine use of kilovoltage cone‐beam computed tomography (kV‐CBCT) imaging in radiation therapy means that assessing image quality is essential for accurate patient set up and delivery of radiation. Unlike diagnostic imaging, the metrics for image quality assessment tools used in radiation therapy are not yet well defined. General recommendations have been made by the AAPM in task group reports such as TG‐142, TG‐179, and most recently TG‐198, concerning the criteria in which a CBCT QA test is within tolerance. However, many of the tolerances often refer to a baseline value determined at the time of commissioning. Therefore, baselines can potentially vary significantly from facility to facility, and even from one machine to another. As a result, current practices for CBCT image QA rely on the methods employed by a particular facility and its medical physicists.

During linear accelerator (LINAC) commissioning, CBCT image quality assessments result in baseline values for the quantity of interest. Monthly checks include image quality tests and, except for geometric distortion, have a recommended value of “baseline” per AAPM TG‐142. AAPM TG‐179 provides more in‐depth QA recommendations regarding CBCT monthly image quality, setting a tolerance of ≤2 mm or 5 lp/cm for high contrast spatial resolution, with other imaging parameters retaining the “baseline” tolerance recommendation.^1^, ^2^ Recently published TG‐198 further refines the monthly CBCT QA recommendations set by previous reports, describing the objective behind each test, tolerances, methods, equipment, and even the estimated amount of time needed for each task.^3^ Additionally, the report makes quantitative changes to tolerances, with spatial resolution returning to baseline, Hounsfield Unit (HU) constancy tolerance set to ±40 HU from those obtained in acceptance, and maintaining a baseline value for the rest of the QA tasks.

Because non‐universal baseline values are used for many monthly CBCT QA metrics, individuals, be it qualified medical physicists or their designated personnel, remain responsible for quantifying both the baselines, and the QA results. Differences can arise between centers’ CBCT QA passing criteria. What is visually ascertained during QA can vary from person to person. Furthermore, different facilities may use a variety of QA devices, have different staffing levels, staff experience, or limited time available for QA. Attempts have been made to fully automate the process of CBCT imaging QA analysis after image acquisition. An approach tested on Varian LINACS with a CATPHAN 500 phantom and Total QA Software (Image Owl, Inc., Greenwich, NY) yielded less‐than‐ideal results due to the software's inability to detect artifacts easily detectable by a human, but not typically sought by the program's QA parameters.^4^ Another method using a Halcyon ring‐gantry LINAC with a QUART phantom (GmbH, Zorneding, Germany) has been more successful.^5^ For C‐arm LINACs, semi‐automatic analysis using a commercial software package allows quick and consistent analysis of key image parameters, irrespective of user dependency. The present work compares visual versus semi‐automatic imaging QA assessments using a commercial image quality assessment software package in combination with a commercially available CT image quality phantom.

## 2 METHODS

The image quality assessments were performed by scanning a Catphan**®** 504 phantom (The Phantom Laboratory, Salem, NY) using a Varian TrueBeam**®** linear accelerator (Varian Medical Systems, Palo Alto, CA). Image analysis was done either visually or using a software package (RIT Radia, Radiological Imaging Technology, Colorado Springs, CO). The evaluation period was 68 months.

Monthly phantom scanning was performed during routine QA using the pelvis imaging protocol (125 kVp, 1080 mAs, with a 2.0 mm slice thickness). This phantom has multiple modules to assess various image quality metrics including high and low contrast resolution (CTP 528 and 515), uniformity and noise (CTP 486), and spatial linearity and Hounsfield Unit constancy (CTP 404).^6^ For the visual method, the values for each metric as detected by either a staff physicist or senior resident were determined and recorded at the TrueBeam XI monitor console immediately after image acquisition by using the available tools. For example, measurement of Hounsfield Unit constancy was done visually by drawing regions of interest on the CTP 404 image to obtain the mean and standard deviation values for each one of the material plugs. For the semi‐automatic method, the DICOM images of the phantom were exported and later evaluated using RIT. The outcomes of these two methods were then compared using statistical analysis.

### 2.1 RIT complete imaging QA setup

Images acquired from scanning the Catphan**®** 504 phantom require setting of specific parameters to ensure proper analysis. Although RIT allows users to use many customized settings, a few general parameters that need adjusting within the “Radia Sequence Editor” are “Type”, “Offset”, and “Analysis” as discussed in the Radia user's manual. Specific settings may vary depending on the phantom used, imaging protocol, image export flow, etc.

**FIGURE 1:**
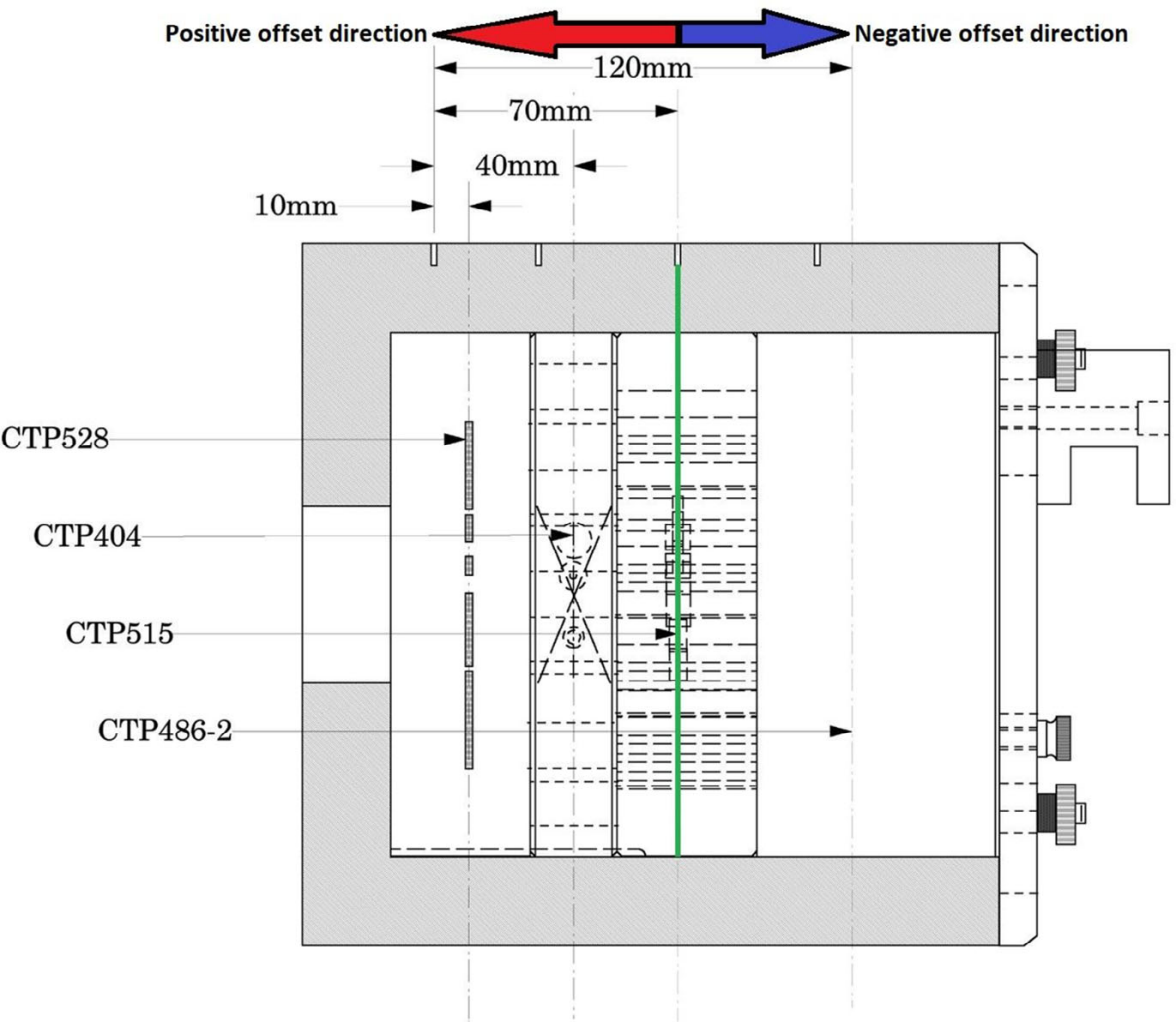
An illustration of the Catphan 504 phantom. The green line represents the reference position used in this study.

A relative offset type was used for analysis since the absolute setting often missed the intended target slice or slices. The Catphan 504 Manual defines distances between modules by using the middle of the CTP 404 module as the zero point or reference position. Our study used CTP 515 instance number (i.e., slice number) 42 as the reference position for relative offset which corresponds to central region of the low contrast resolution module. From the reference position, the target analysis slices were set to −50 mm offset for the uniformity module, +32 mm offset for the Hounsfield unit and linearity module, and +64 mm offset for the spatial resolution module. Figure 1 shows the reference position and offset directionality.

#### 2.1.1 High contrast resolution analysis

Module 528 instance number 74 was analyzed. The “Low Value Gap” was changed from the default of 6 when analysis resulted in misplacement of the locations of each line pair grouping on the image.

#### 2.1.2 Low contrast resolution analysis

Inherently low contrast objects can be challenging for both users and software to detect due to low signal‐to‐noise ratio images. One way to increase low contrast disk detectability within CTP 515 with RIT is to average multiple slices to create a composite image. RIT provides an averaging solution for this module for which the maximum setting of seven, meaning seven averaged slices, was chosen around instance number 42. The resulting slice used for analysis is therefore an average of the central slice (instance number 42), the three preceding, and the three succeeding slices. Adjusting the “Trim Margin”, which determines the radius of the image analyzed by the software, is then done so the analysis region of interest runs through the center of the largest contrast object.^7^ A “Trim Margin” of 11 was selected with the large contrast object position set to the top of the image.

#### 2.1.3 Hounsfield unit constancy analysis

Setting up the CTP 404 module's Hounsfield unit component did not require adjusting any of the default RIT settings for correct analysis. RIT permits changes to the expected CT# values by changing the “Estimated Background CT Number” (default of 100), or the “Estimated Air CT Number” (−1000) if the software cannot correctly identify materials. The software's pass/fail criteria can also be adjusted by the user if desired by adjusting the minimum and maximum CT number value thresholds. Instance number 59 was chosen for analysis except for when a given month's data had the module's center in a different instance number.

#### 2.1.4 Uniformity and noise analysis

Instance number 19 was selected for analysis and determined from the relative distances between modules. None of the default RIT values were altered, with a default value of ±30 HU compared to the mean HU value of the five regions of interest serving as the software's pass/fail criteria. The default “Profile Filter Size” of 15 was utilized, which controls how “smooth” the resulting vertical, and horizontal HU non‐uniformity integral profiles are in the analysis report. According to TG‐179, both uniformity and noise are characterized by the variability of the average signal over several small regions of interest.^2^ Therefore, the noise can be characterized by the maximum average standard deviation HU value of the ROI profiles created by RIT.

#### 2.1.5 Spatial linearity analysis

For the CTP 404 module, RIT uses a pass/fail criteria of ±0.04 mm by default, which is more constrained than the visual method's 1 mm threshold from the expected 50 mm. The two relevant settings which can be adjusted are the “Teflon Rod Threshold”, and the “Air Rod Threshold” should the software fail to correctly center the rods, or when there are too many or too few rods detected.^7^ No adjustment of the default settings was necessary. Since module 404 is also used for HU constancy, the same instance number 59 was selected with a few exceptions noted in the following discussion.

### 2.2 Statistical methods

In order to compare the two methods, two statistical analysis methods (Bland Altman and Paired Sample *T*‐Test) were used:

Bland Altman (B.A.) Analysis plots represent the differences between paired data and allow observers to get an idea of paired data behavior at a glance. The spread of the points around the bias, the distribution of mean values, and the limits of agreement (L.O.A.) are used to interpret the plots.

Paired Sample *T*‐Test consists of two hypotheses: the null hypothesis which assumes there is no difference between paired data samples, and the alternative hypothesis which assumes there is a difference. For our purposes, the key values used for analysis are P (one tail) with a value of *P* = 0.05, *t*‐stat, and *t*‐critical, whereby |*t*‐stat| > | *t*‐critical| means there is a statistically significant difference between data obtained from each of the acquisition methods.

## 3 RESULTS

### 3.1 High contrast resolution

A comparison between the two methods of image analysis was made using the two aforementioned statistical analysis methods. Four data points were excluded from the final analysis due to poor image quality despite reducing the low value gap parameter down to 2. Ten data points were analyzed with a different instance number/slice when the image quality was better than that of instance 74, when the DICOM files were disordered, or when the month's data contained a different number of DICOM files than the expected 89.

**FIGURE 2:**
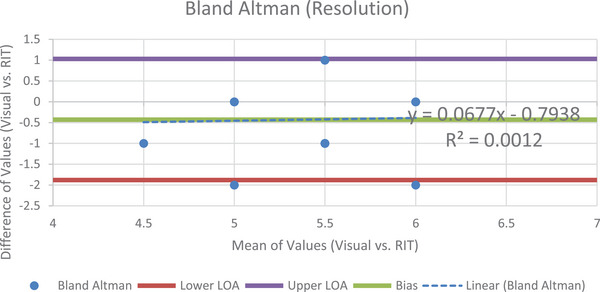
The B.A. plot for the high contrast resolution module.

Slight changes to phantom positioning could explain the variability in image quality between slices, but given that observers used the same images, the effect of this shift on comparisons should be negligible. Figure 2 is the Bland Altman plot for the high contrast resolution module.

**FIGURE 3:**
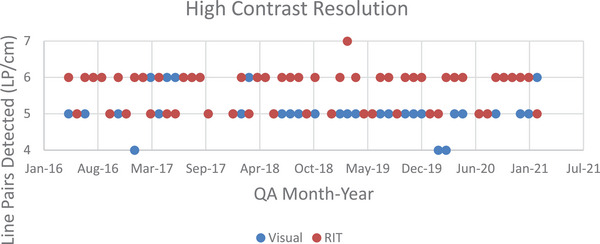
A scatter plot of the line pairs detected by each analysis method over time.

The plot has a bias of −0.43, a lower limit of agreement of −1.88, and an upper limit of agreement of 1.03. As seen in the figure, there is a fairly linear distribution of points with relatively narrow limits of agreement. Another feature is the mean of the values concentrating around 5−6 lp/cm, which with a bias of −0.43, means that the two methods are largely equivalent. However, there is a significant difference between data obtained from each of the acquisition methods indicated by a *t*‐critical value of 1.670, and a t‐stat value of −4.220. Calculating the P value for the high contrast resolution, neither the visual nor the semi‐automatic results occurred by chance (*P* = 4.86E‐05). Figure 3 shows the visual and software‐derived values for the number of line pairs/cm used for high contrast resolution determination.

### 3.2 Low contrast resolution

**FIGURE 4:**
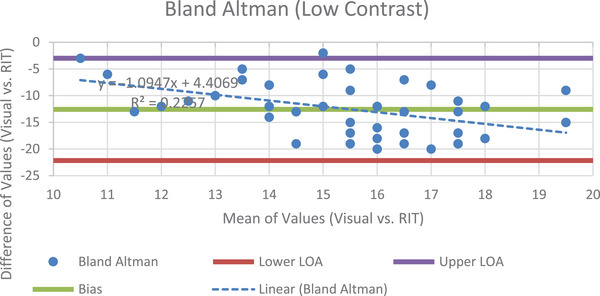
The B.A. plot for the low contrast module.

Visual determination of the number of low contrast circles in a single slice were compared to the software's seven slice average. One month of data used instance 22 for analysis due to a shift required by another module's data being unavailable. Figure 4 shows the Bland Altman analysis of the low contrast resolution.

**FIGURE 5:**
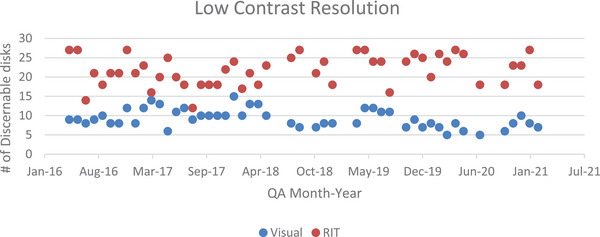
A scatter plot showing the distribution of discernable disks with each method over time.

Two things to note in the plot above are how large the bias and limits of agreements are. With a bias of −12.57, a lower LOA of −22.16, and an upper LOA of −2.99, there is a large difference between values, indicating that visual measurement detected considerably fewer low contrasts disks when compared to semi‐automatic analysis. The one tail *P* value of 2.814E‐23 means that it is virtually impossible that the results obtained occurred by chance. Furthermore, with a *t*‐critical of 1.677 and a *t*‐stat value of −17.994, it is clear that the semi‐automatic results are quite different from those obtained visually. Figure 5 shows the visual and software‐derived values for the number of circles used for low contrast resolution determination.

### 3.3 Hounsfield unit constancy

**FIGURE 6:**
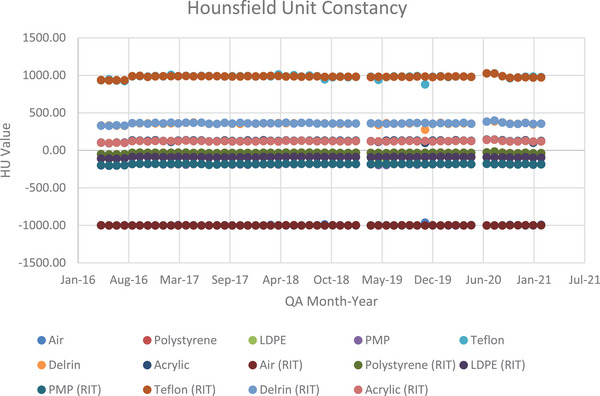
A scatter plot showing the distribution of HU values assigned to each material by each method over time.

Unlike in the last two modules, only a paired *t*‐test analysis was performed for this module due to the large volume of data involving many different materials. Instance number 39 was used for one data point due to the missing module previously mentioned in Section 3.2, instance 62 was used for two others due to a shift in DICOM position, and instance 23 was used for another data point due to DICOM image disorder. The HU values for each of the methods were plotted over time as shown in Figure 6.

Due to the wide range of HU values, it is difficult to make qualitative observations from the figure. Except for Teflon, it can be concluded within a significance level of 5% that the measured results did not occur by chance. Two material analyzes, LDPE, and Teflon, were not significantly different. The reason LDPE and Teflon were not significantly different is not readily apparent.

### 3.4 Uniformity and noise

**FIGURE 7:**
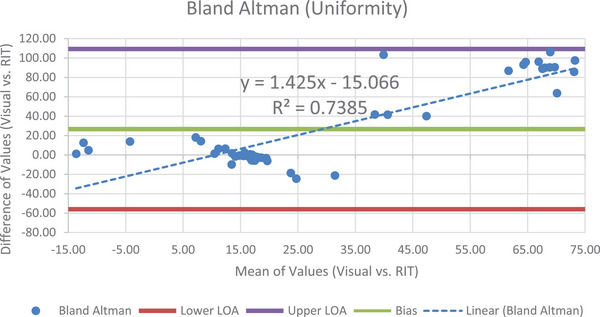
The B.A. plot for the uniformity module, showing two distinct clusters of data points.

Once again, B.A. was used in conjunction with paired t‐test analysis in this module. Three months’ data were excluded from analysis due to the uniformity module being missing in the DICOM images, unusable DICOM files, and lack of a suitable instance. Some months’ data contained visual elements in the analysis slice affecting the uniformity of the image such as darker or brighter regions. However, these images were included since an analysis objective was to consistently use the same instance for semi‐automated data collection, especially in this module where the exact instance number/slice used by each of the visual observers is unknown. Seen in Figure 7 is the B.A. plot for CTP 486.

An interesting feature of this plot is the concentration of points along different regions, with two clusters appearing to coincide more closely in mean value and difference of values. One possible explanation for this could be the way two or more observers evaluated or selected the image for analysis. There are also wide limits of agreements of −56.01 for the lower LOA, and 109.29 for the upper LOA. The bias of 26.64 HU, combined with the software analysis reporting values more closely aligned with the expected value of ±30 HU set by RIT indicate a difference between the two methods of data collection. Statistically, the assessment of dissimilarity between analysis methods is supported (one tail *P* value = 3.331E‐05, *t*‐stat value = 4.377, *t*‐critical value = 1.678).

RIT's analysis based on five ROIs allows noise to be characterized across the image. The visual method for uniformity analysis however considered only one large ROI providing the average, and standard deviation of the pixel values within. An example considering only the average standard deviations between these two methods for 2017 alone provides 5.36 HU using RIT, and 3.42 HU using the visual method.

### 3.5 Spatial linearity

Both visual and semi‐automatic methods of analysis provided closely matching values; therefore, a comparison of the average differences between rod distances was made. The same slices were used as in the HU constancy analysis, as analysis was performed for both module portions at once. Based on the largest average change between the measured distances via both analysis methods being a maximum of 0.17%, it is clear that each method recorded similar distances on average.

## 4 DISCUSSION

Characterizing automated CBCT QA methods is an area of active research. Manger et al's automated QA solution demonstrated that meeting or exceeding task group and/or vendor recommendations for passing QA criteria can still result in image quality issues going undetected by fully automated software. The approach taken used uniformity as the best predictor of CBCT imaging issues. One such instance had the prior day's software QA exceeding the QC passing criteria for spatial resolution, geometric distortion, contrast, and uniformity yet presented streaking artifacts during a patient scan which were ignored by the software during the troubleshooting scan. Since software‐based QA focuses on regions of interest, it can easily overlook aspects outside its base parameters that are otherwise easily discernable. Semi‐automated QA software also focuses on regions of interest; therefore, without a physicist visually confirming the automated portion of the QA, semi‐automated QA has the same shortcomings.

Another fully automated software was developed and used for kV CBCT imaging QA on a Halcyon unit over a period of 10 months (Peng J et al). The software was able to provide results meeting or exceeding manufacturer recommendations and generally accepted standards. Furthermore, the analysis of 19 protocols was completed in about two minutes, highlighting the time advantages of automated QA. Comparison between our study using RIT software and their approach is primarily limited by the differences in LINAC and phantom designs used. Another caveat is that the phantom used in that study (QUART phantom) lacks an obvious, visually discernable module component for low and high contrast resolution, which exists in the Catphan phantom. Low contrast analysis uses SNR and CNR, while high contrast analysis uses edge‐profile MTF. While no imaging artifacts or other inconveniences were reported, it is unknown how their software would react to, and grade such occurrences. Furthermore, the absence of a dedicated, observable analysis marker could hinder visual troubleshooting of the low and high contrast modules if automated QA fails. The authors are working on extending their study to other LINACs at multiple institutions.

Other automated imaging QA software tools are available for physicists to use. Pylinac, an open‐source Python programming tool, provides scripts for automated image analysis of protocols like TG‐142 which can be customized to the users’ needs. Varian's DoseLab contains multiple QA tools for simplified automation with trend analysis that can be assigned to specific users. IBA's (IBA Dosimetry, Schwarzenbruck, Germany) 3D image quality plug‐in for myQA machines offers QA‐based generic and customizable protocols for comprehensive imaging QA analysis. More QA software tools exist or are in development, which means that automated or semi‐automated QA will likely continue to increase in prevalence over time.

Regarding the benefits of automated image quality assessment, it is worth noting the consistency of automated results over visual ones, the removal of user dependency in analysis, and the potentially time savings. Increased consistency in the image analysis also aids in setting baselines and comparing various systems without the biases of individuals performing the tests visually. Other benefits of automation include allowing less experienced users to perform the image quality tests, and tracking the image quality changes over time.

## 5 CONCLUSION

Visual CBCT QA was compared to semi‐automatic software analysis in this study. The results indicate greater stability using semi‐automatic software analysis compared to visual measurements, especially for low/high contrast resolution, as well as uniformity. Furthermore, the results obtained match or exceed visual QA passing criteria. Once the software setup is performed, semi‐automated CBCT image QA offers a convenient means to quantify monthly QA metrics which can be measured with relative ease and speed.

## AUTHOR CONTRIBUTIONS

Nicholas Becerra‐Espinosa, Lindsey Claps, and Parham Alaei conceived and designed the project. Lindsey Claps and Parham Alaei acquired the data. Nicholas Becerra‐Espinosa, Lindsey Claps, and Parham Alaei analyzed and interpreted the data. Nicholas Becerra‐Espinosa, Lindsey Claps, and Parham Alaei wrote the paper.

## CONFLICT OF INTERESTS STATEMENT

The authors declare no conflicts of interest.

## Data Availability

Data available upon reasonable request.
